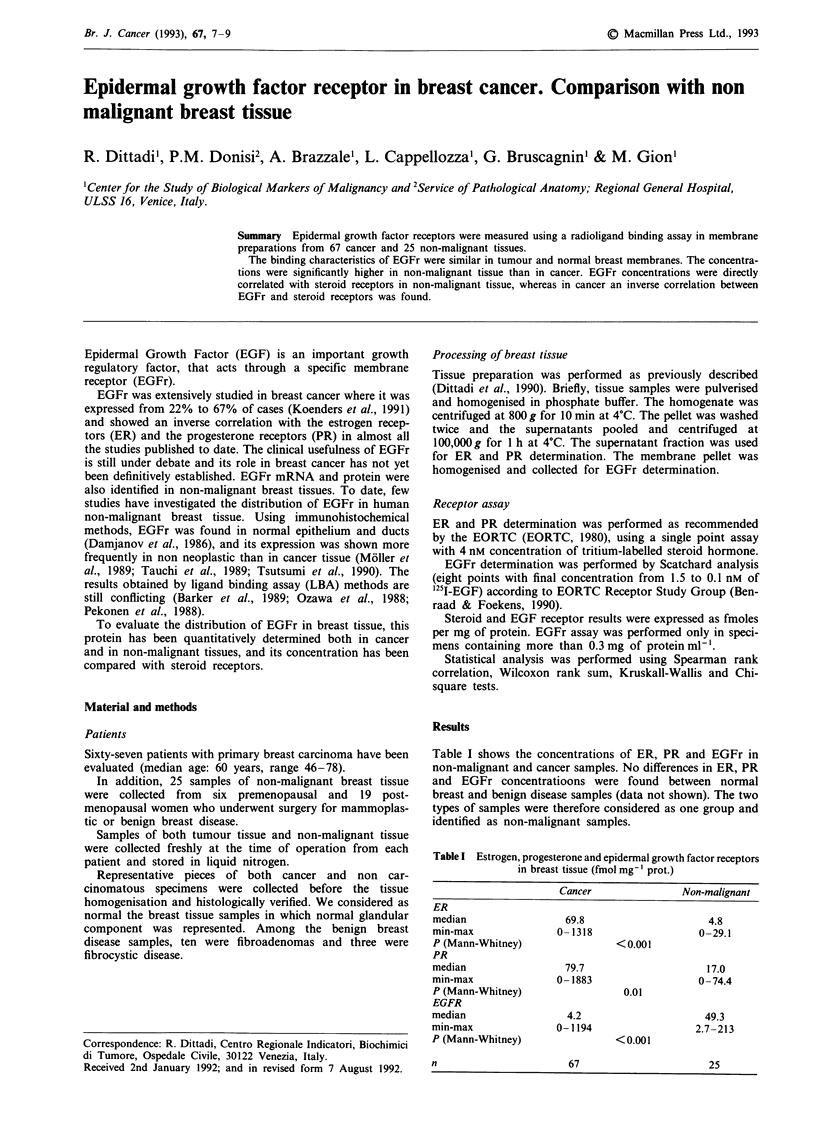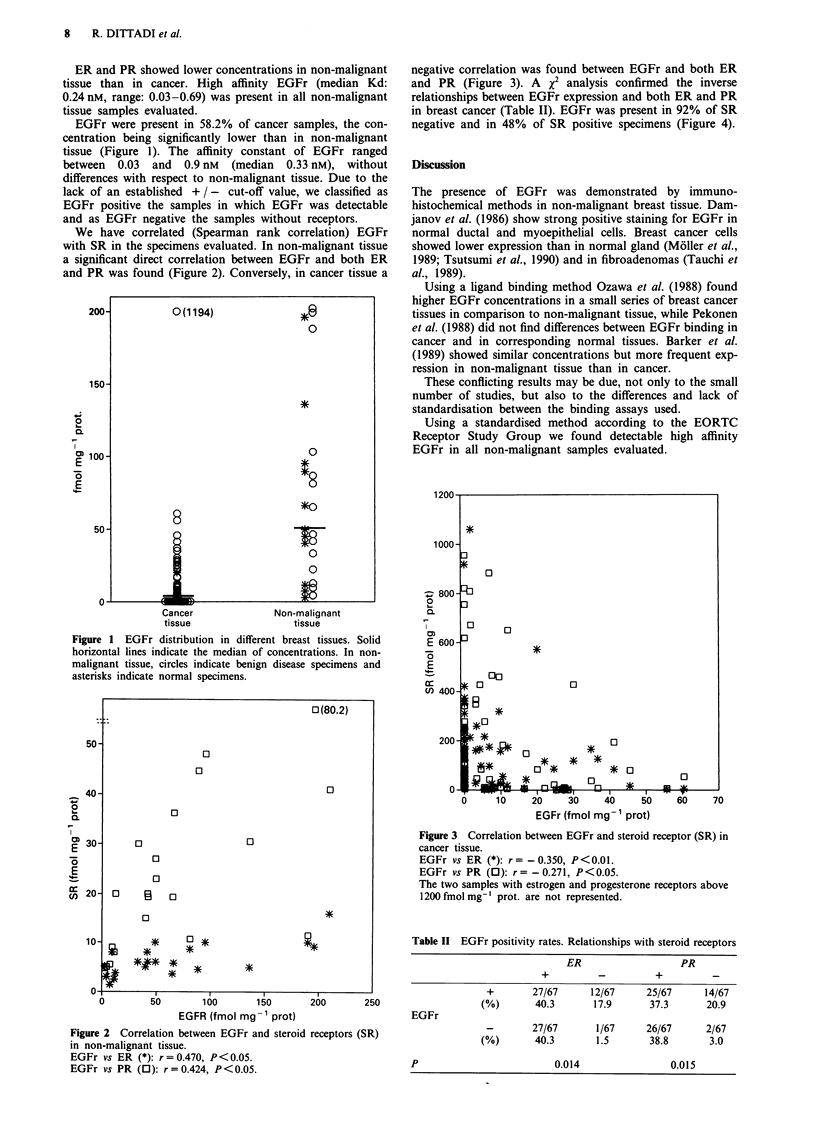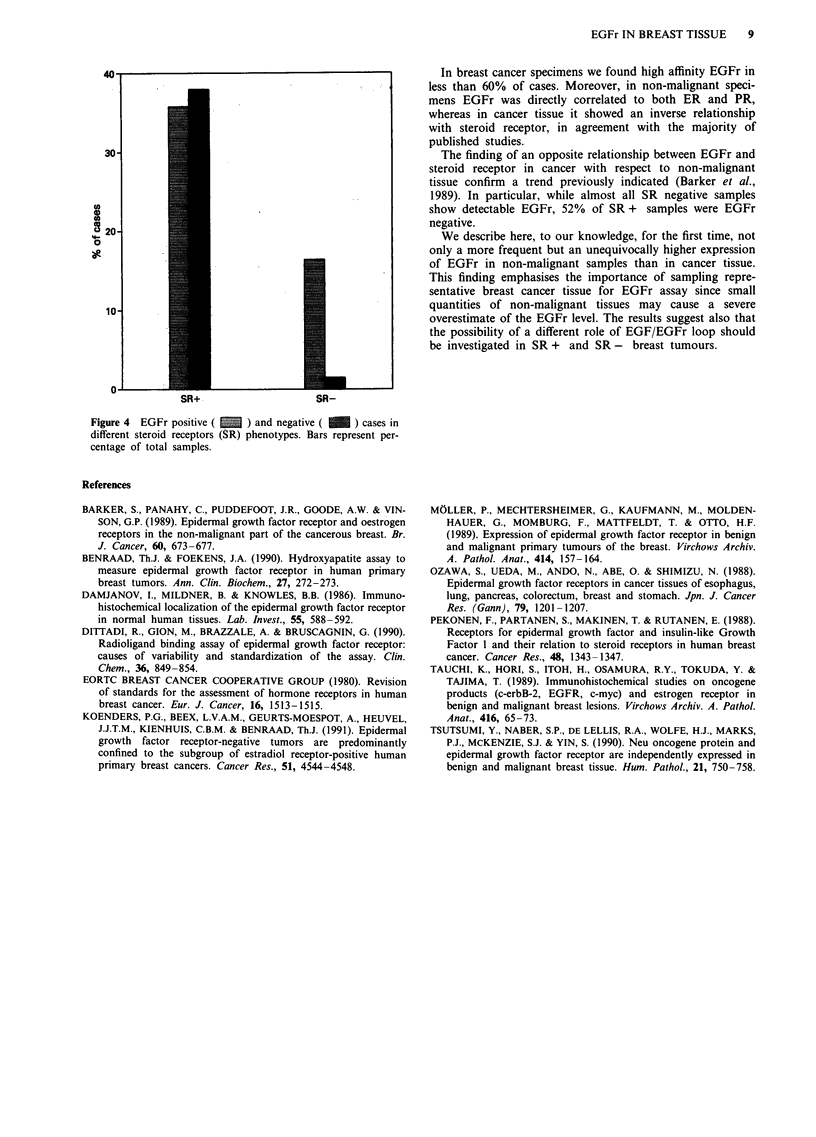# Epidermal growth factor receptor in breast cancer. Comparison with non malignant breast tissue.

**DOI:** 10.1038/bjc.1993.2

**Published:** 1993-01

**Authors:** R. Dittadi, P. M. Donisi, A. Brazzale, L. Cappellozza, G. Bruscagnin, M. Gion

**Affiliations:** Center for the Study of Biological Markers of Malignancy, Regional General Hospital, Venice, Italy.

## Abstract

Epidermal growth factor receptors were measured using a radioligand binding assay in membrane preparations from 67 cancer and 25 non-malignant tissues. The binding characteristics of EGFr were similar in tumour and normal breast membranes. The concentrations were significantly higher in non-malignant tissue than in cancer. EGFr concentrations were directly correlated with steroid receptors in non-malignant tissue, whereas in cancer an inverse correlation between EGFr and steroid receptors was found.


### Epidermal growth factor receptor in breast cancer. Comparison with non malignant breast tissue

R. Dittadi[1], P.M. Donisi[2], A. Brazzale[1], L. Cappellozza[1], G. Bruscagnin[1] & M. Gion[1]

[1]*Center for the Study of Biological Markers of Malignancy and* [2]*Service of Pathological Anatomy; Regional General Hospital, ULSS 16, Venice, Italy.*

**Summary** Epidermal growth factor receptors were measured using a radioligand binding assay in membrane preparations from 67 cancer and 25 non-malignant tissues.

The binding characteristics of EGFr were similar in tumour and normal breast membranes. The concentrations were significantly higher in non-malignant tissue than in cancer. EGFr concentrations were directly correlated with steroid receptors in non-malignant tissue, whereas in cancer an inverse correlation between EGFr and steroid receptors was found.

Epidermal Growth Factor (EGF) is an important growth regulatory factor, that acts through a specific membrane receptor (EGFr).

EGFr was extensively studied in breast cancer where it was expressed from 22% to 67% of cases (Koenders *et al.*, 1991) and showed an inverse correlation with the estrogen receptors (ER) and the progesterone receptors (PR) in almost all the studies published to date. The clinical usefulness of EGFr is still under debate and its role in breast cancer has not yet been definitively established. EGFr mRNA and protein were also identified in non-malignant breast tissues. To date, few studies have investigated the distribution of EGFr in human non-malignant breast tissue. Using immunohistochemical methods, EGFr was found in normal epithelium and ducts (Damjanov *et al.*, 1986), and its expression was shown more frequently in non neoplastic than in cancer tissue (Möller *et al.*, 1989; Tauchi *et al.*, 1989; Tsutsumi *et al.*, 1990). The results obtained by ligand binding assay (LBA) methods are still conflicting (Barker *et al.*, 1989; Ozawa *et al.*, 1988; Pekonen *et al.*, 1988).

To evaluate the distribution of EGFr in breast tissue, this protein has been quantitatively determined both in cancer and in non-malignant tissues, and its concentration has been compared with steroid receptors.

### Material and methods

### *Patients*

Sixty-seven patients with primary breast carcinoma have been evaluated (median age: 60 years, range 46–78).

In addition, 25 samples of non-malignant breast tissue were collected from six premenopausal and 19 post-menopausal women who underwent surgery for mammoplastic or benign breast disease.

Samples of both tumour tissue and non-malignant tissue were collected freshly at the time of operation from each patient and stored in liquid nitrogen.

Representative pieces of both cancer and non carcinomatous specimens were collected before the tissue homogenisation and histologically verified. We considered as normal the breast tissue samples in which normal glandular component was represented. Among the benign breast disease samples, ten were fibroadenomas and three were fibrocystic disease.

Correspondence: R. Dittadi, Centro Regionale Indicatori, Biochimici di Tumore, Ospedale Civile, 30122 Venezia, Italy.
Received 2nd January 1992; and in revised form 7 August 1992.

### *Processing of breast tissue*

Tissue preparation was performed as previously described (Dittadi *et al.*, 1990). Briefly, tissue samples were pulverised and homogenised in phosphate buffer. The homogenate was centrifuged at 800 *g* for 10 min at 4°C. The pellet was washed twice and the supernatants pooled and centrifuged at 100,000 *g* for 1 h at 4°C. The supernatant fraction was used for ER and PR determination. The membrane pellet was homogenised and collected for EGFr determination.

### *Receptor assay*

ER and PR determination was performed as recommended by the EORTC (EORTC, 1980), using a single point assay with 4 nM concentration of tritium-labelled steroid hormone.

EGFr determination was performed by Scatchard analysis (eight points with final concentration from 1.5 to 0.1 nM of $^{125}$I-EGF) according to EORTC Receptor Study Group (Benraad & Foekens, 1990).

Steroid and EGF receptor results were expressed as fmoles per mg of protein. EGFr assay was performed only in specimens containing more than 0.3 mg of protein $ml^{-1}$.

Statistical analysis was performed using Spearman rank correlation, Wilcoxon rank sum, Kruskall-Wallis and Chi-square tests.

### Results

Table I shows the concentrations of ER, PR and EGFr in non-malignant and cancer samples. No differences in ER, PR and EGFr concentratioons were found between normal breast and benign disease samples (data not shown). The two types of samples were therefore considered as one group and identified as non-malignant samples.

**Table I** Estrogen, progesterone and epidermal growth factor receptors in breast tissue (fmol $mg^{-1}$ prot.)

| | *Cancer* | | *Non-malignant* |
|---|---|---|---|
| *ER* | | | |
| median | 69.8 | | 4.8 |
| min-max | 0–1318 | | 0–29.1 |
| *P* (Mann-Whitney) | | <0.001 | |
| *PR* | | | |
| median | 79.7 | | 17.0 |
| min-max | 0–1883 | | 0–74.4 |
| *P* (Mann-Whitney) | | 0.01 | |
| *EGFR* | | | |
| median | 4.2 | | 49.3 |
| min-max | 0–1194 | | 2.7–213 |
| *P* (Mann-Whitney) | | <0.001 | |
| *n* | 67 | | 25 |

ER and PR showed lower concentrations in non-malignant tissue than in cancer. High affinity EGFr (median Kd: 0.24 nM, range: 0.03–0.69) was present in all non-malignant tissue samples evaluated.

EGFr were present in 58.2% of cancer samples, the concentration being significantly lower than in non-malignant tissue (Figure 1). The affinity constant of EGFr ranged between 0.03 and 0.9 nM (median 0.33 nM), without differences with respect to non-malignant tissue. Due to the lack of an established +/− cut-off value, we classified as EGFr positive the samples in which EGFr was detectable and as EGFr negative the samples without receptors.

We have correlated (Spearman rank correlation) EGFr with SR in the specimens evaluated. In non-malignant tissue a significant direct correlation between EGFr and both ER and PR was found (Figure 2). Conversely, in cancer tissue a negative correlation was found between EGFr and both ER and PR (Figure 3). A $\chi^2$ analysis confirmed the inverse relationships between EGFr expression and both ER and PR in breast cancer (Table II). EGFr was present in 92% of SR negative and in 48% of SR positive specimens (Figure 4).

**Figure 1** EGFr distribution in different breast tissues. Solid horizontal lines indicate the median of concentrations. In non-malignant tissue, circles indicate benign disease specimens and asterisks indicate normal specimens.

**Figure 2** Correlation between EGFr and steroid receptors (SR) in non-malignant tissue.
EGFr *vs* ER (*): $r = 0.470$, $P < 0.05$.
EGFr *vs* PR (□): $r = 0.424$, $P < 0.05$.

### Discussion

The presence of EGFr was demonstrated by immunohistochemical methods in non-malignant breast tissue. Damjanov *et al.* (1986) show strong positive staining for EGFr in normal ductal and myoepithelial cells. Breast cancer cells showed lower expression than in normal gland (Möller *et al.*, 1989; Tsutsumi *et al.*, 1990) and in fibroadenomas (Tauchi *et al.*, 1989).

Using a ligand binding method Ozawa *et al.* (1988) found higher EGFr concentrations in a small series of breast cancer tissues in comparison to non-malignant tissue, while Pekonen *et al.* (1988) did not find differences between EGFr binding in cancer and in corresponding normal tissues. Barker *et al.* (1989) showed similar concentrations but more frequent expression in non-malignant tissue than in cancer.

These conflicting results may be due, not only to the small number of studies, but also to the differences and lack of standardisation between the binding assays used.

Using a standardised method according to the EORTC Receptor Study Group we found detectable high affinity EGFr in all non-malignant samples evaluated.

**Figure 3** Correlation between EGFr and steroid receptor (SR) in cancer tissue.
EGFr *vs* ER (*): $r = -0.350$, $P < 0.01$.
EGFr *vs* PR (□): $r = -0.271$, $P < 0.05$.
The two samples with estrogen and progesterone receptors above 1200 fmol mg$^{-1}$ prot. are not represented.

**Table II** EGFr positivity rates. Relationships with steroid receptors

| | | *ER* + | *ER* − | *PR* + | *PR* − |
|---|---|---|---|---|---|
| EGFr | + | 27/67 | 12/67 | 25/67 | 14/67 |
| | (%) | 40.3 | 17.9 | 37.3 | 20.9 |
| | − | 27/67 | 1/67 | 26/67 | 2/67 |
| | (%) | 40.3 | 1.5 | 38.8 | 3.0 |
| *P* | | 0.014 | | 0.015 | |

**Figure 4** EGFr positive ( ▒ ) and negative ( ▓ ) cases in different steroid receptors (SR) phenotypes. Bars represent percentage of total samples.

In breast cancer specimens we found high affinity EGFr in less than 60% of cases. Moreover, in non-malignant specimens EGFr was directly correlated to both ER and PR, whereas in cancer tissue it showed an inverse relationship with steroid receptor, in agreement with the majority of published studies.

The finding of an opposite relationship between EGFr and steroid receptor in cancer with respect to non-malignant tissue confirm a trend previously indicated (Barker *et al.*, 1989). In particular, while almost all SR negative samples show detectable EGFr, 52% of SR + samples were EGFr negative.

We describe here, to our knowledge, for the first time, not only a more frequent but an unequivocally higher expression of EGFr in non-malignant samples than in cancer tissue. This finding emphasises the importance of sampling representative breast cancer tissue for EGFr assay since small quantities of non-malignant tissues may cause a severe overestimate of the EGFr level. The results suggest also that the possibility of a different role of EGF/EGFr loop should be investigated in SR + and SR − breast tumours.

### References

BARKER, S., PANAHY, C., PUDDEFOOT, J.R., GOODE, A.W. & VINSON, G.P. (1989). Epidermal growth factor receptor and oestrogen receptors in the non-malignant part of the cancerous breast. *Br. J. Cancer*, **60**, 673–677.

BENRAAD, Th.J. & FOEKENS, J.A. (1990). Hydroxyapatite assay to measure epidermal growth factor receptor in human primary breast tumors. *Ann. Clin. Biochem.*, **27**, 272–273.

DAMJANOV, I., MILDNER, B. & KNOWLES, B.B. (1986). Immunohistochemical localization of the epidermal growth factor receptor in normal human tissues. *Lab. Invest.*, **55**, 588–592.

DITTADI, R., GION, M., BRAZZALE, A. & BRUSCAGNIN, G. (1990). Radioligand binding assay of epidermal growth factor receptor: causes of variability and standardization of the assay. *Clin. Chem.*, **36**, 849–854.

EORTC BREAST CANCER COOPERATIVE GROUP (1980). Revision of standards for the assessment of hormone receptors in human breast cancer. *Eur. J. Cancer*, **16**, 1513–1515.

KOENDERS, P.G., BEEX, L.V.A.M., GEURTS-MOESPOT, A., HEUVEL, J.J.T.M., KIENHUIS, C.B.M. & BENRAAD, Th.J. (1991). Epidermal growth factor receptor-negative tumors are predominantly confined to the subgroup of estradiol receptor-positive human primary breast cancers. *Cancer Res.*, **51**, 4544–4548.

MÖLLER, P., MECHTERSHEIMER, G., KAUFMANN, M., MOLDENHAUER, G., MOMBURG, F., MATTFELDT, T. & OTTO, H.F. (1989). Expression of epidermal growth factor receptor in benign and malignant primary tumours of the breast. *Virchows Archiv. A. Pathol. Anat.*, **414**, 157–164.

OZAWA, S., UEDA, M., ANDO, N., ABE, O. & SHIMIZU, N. (1988). Epidermal growth factor receptors in cancer tissues of esophagus, lung, pancreas, colorectum, breast and stomach. *Jpn. J. Cancer Res. (Gann)*, **79**, 1201–1207.

PEKONEN, F., PARTANEN, S., MAKINEN, T. & RUTANEN, E. (1988). Receptors for epidermal growth factor and insulin-like Growth Factor 1 and their relation to steroid receptors in human breast cancer. *Cancer Res.*, **48**, 1343–1347.

TAUCHI, K., HORI, S., ITOH, H., OSAMURA, R.Y., TOKUDA, Y. & TAJIMA, T. (1989). Immunohistochemical studies on oncogene products (c-erbB-2, EGFR, c-myc) and estrogen receptor in benign and malignant breast lesions. *Virchows Archiv. A. Pathol. Anat.*, **416**, 65–73.

TSUTSUMI, Y., NABER, S.P., DE LELLIS, R.A., WOLFE, H.J., MARKS, P.J., MCKENZIE, S.J. & YIN, S. (1990). Neu oncogene protein and epidermal growth factor receptor are independently expressed in benign and malignant breast tissue. *Hum. Pathol.*, **21**, 750–758.